# Obesity-induced pyroptotic adipocyte death leads to TREM2-dependent macrophage dysfunction and adipose tissue inflammation

**DOI:** 10.1016/j.isci.2025.114358

**Published:** 2025-12-06

**Authors:** Cheoljun Choi, Junhyuck Lee, Gyeongran Park, Sik Namgoong, Yun-Hee Lee

**Affiliations:** 1 College of Pharmacy and Research Institute of Pharmaceutical Sciences, Seoul National University, Seoul 08826, Republic of Korea; 2 Department of Plastic Surgery, Korea University College of Medicine, Seoul, Republic of Korea

**Keywords:** molecular interaction, molecular network, immune response

## Abstract

Triggering receptor expressed on myeloid cells 2 (TREM2) is a key marker of lipid-associated macrophages (LAMs), but its role in adipose tissue homeostasis remains unclear due to conflicting results. This study aimed to investigate the role of TREM2 in adipose tissue inflammation and metabolic dysfunction during high-fat diet (HFD)–induced obesity. HFD feeding enhanced proteolytic cleavage of TREM2 in gonadal white adipose tissue (GWAT), driven by upregulation of the metalloproteinase ADAM10 and ADAM17. *In vitro* co-culture of RAW264.7 cells with pyroptotic, but not apoptotic, adipocytes increased stimulator of interferon genes (STING) activation, upregulated ADAM10/17 expression, and promoted TREM2 shedding. Pharmacological inhibition of ADAM10/17 by GM6001 reduced TREM2 cleavage and enhanced phagocytosis of dying pyroptotic adipocytes. *In vivo* GM6001 treatment attenuated HFD-induced weight gain, improved metabolic parameters, and shifted macrophage polarization toward anti-inflammatory TREM2+CD206+ subsets. These findings demonstrate that pyroptotic adipocyte death promotes pathological TREM2 shedding, contributing to macrophage dysfunction and adipose tissue inflammation.

## Highlights

• HFD increases TREM2 expression and cleavage in gonadal adipose tissue of mice
• Pyroptotic adipocytes activate STING-ADAM10/17 axis and induce TREM2 shedding
• GM6001 reduces TREM2 shedding and enhances macrophage phagocytosis
• GM6001 improves obesity-induced inflammation and metabolic dysfunction

## Graphical abstract

**Figure:**
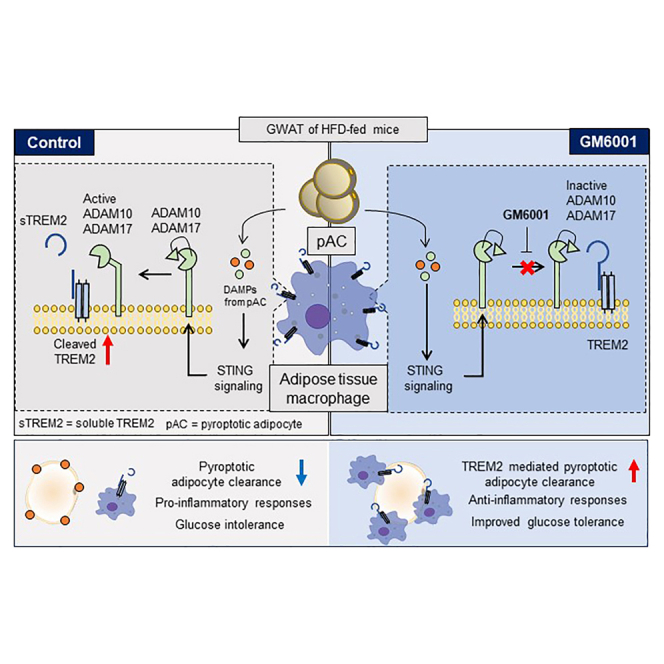

## Introduction

Obesity drives pathological remodeling of adipose tissue, marked by adipocyte hypertrophy, chronic inflammation, and insulin resistance.^1^ Among the initiating factors, obesity-induced adipocyte death has been implicated as a key trigger that promotes the recruitment of pro-inflammatory adipose tissue macrophages. This, in turn, contributes to systemic metabolic impairments, such as glucose intolerance and insulin resistance.^2^^,^^3^ Recent studies have identified lipid-associated macrophages (LAMs) as a distinct macrophage subset characterized by the expression of triggering receptor expressed on myeloid cells 2 (TREM2). These macrophages are recruited to adipose tissue in response to lipid overload under pathological conditions such as obesity.^4^^,^^5^^,^^6^ Similarly, TREM2-positive macrophages are also enriched in the liver during metabolic dysfunction-associated steatohepatitis (MASH)^7^^,^^8^ and in foam cells within atherosclerotic lesions.^9^ Numerous studies have reported that obesity-induced LAMs appear to express the genes associated with pro-inflammatory cytokines,^5^ chemokines,^10^ and metabolic activation.^11^

Although TREM2-positive LAMs are implicated in lipid metabolic processes, the precise roles of these macrophages in adipose tissue remain incompletely understood. Our previous observation demonstrated that a high-fat diet (HFD) induces TREM2-positive macrophages in gonadal white adipose tissue (GWAT) that exhibit pro-inflammatory phenotypes.^6^ Consistently, in both obese mice and patients with obesity, the accumulation of TREM2-positive, LAMs has been linked to impaired clearance of dead adipocytes and the progression of insulin resistance and metabolic dysfunction.^11^^,^^12^ However, prior studies have reported that both gain- and loss-of-function of TREM2 lead to increased HFD-induced weight gain, adipocyte hypertrophy, and insulin resistance in mice.^4^^,^^5^ These conflicting findings suggest that the functional role of TREM2-positive macrophage recruitment in adipose tissue, whether protective or detrimental, remains unresolved.

Multiple studies have linked TREM2 mutations and genetic variants to an elevated risk of neurodegenerative disorders, including Alzheimer’s disease (AD),^13^^,^^14^ Parkinson’s disease,^15^ and frontotemporal lobar degeneration (FTLD)-like syndrome.^16^ The TREM2 signaling in microglia regulates lipid sensing, facilitates amyloid plaque containment, promotes microglial proliferation, suppresses neuro-inflammation, and enhances phagocytic activity.^17^^,^^18^ However, in AD, TREM2 undergoes proteolytic cleavage by a disintegrin and metalloproteases 10/17 (ADAM10/17) between residues His^157^ and Ser^158^, releasing a soluble fragment (sTREM2) detectable in cerebrospinal fluid.^19^ Elevated sTREM2 levels can be observed near necrotic neurons and abnormal protein aggregates,^20^ and circulating sTREM2 can also enter the brain parenchyma and exacerbate neurodegenerative pathology.^21^ Paradoxically, transgenic mice with reduced TREM2 shedding exhibit worsened neuro-inflammation in the context of amyloid-β pathology, highlighting a complex role of TREM2 cleavage in modulating inflammatory responses.^22^ Likewise, recent studies have further shown that increased sTREM2 and TREM2 shedding are associated with hepatic lipid accumulation and the progression of MASH^7^^,^^23^ and chronic liver diseases.^24^ Despite these findings, the role and consequences of TREM2 shedding in adipose tissue remain poorly understood.

In this study, we hypothesize that HFD feeding increases TREM2 shedding and contribute to adipose tissue inflammation in GWAT. Specifically, we propose that enhanced cleavage of TREM2 exacerbates HFD-induced inflammatory responses and metabolic dysfunction. To test this hypothesis, we examined whether pharmacological inhibition of ADAM proteases responsible for TREM2 shedding can mitigate HFD-induced adipose tissue inflammation and restore metabolic homeostasis. This study aims to elucidate the functional role of TREM2 cleavage in adipose tissue and explore its potential as a therapeutic target for obesity-related metabolic disorders.

## Results

### High-fat diet feeding increases cleavage of TREM2 in GWAT

**Figure 1:**
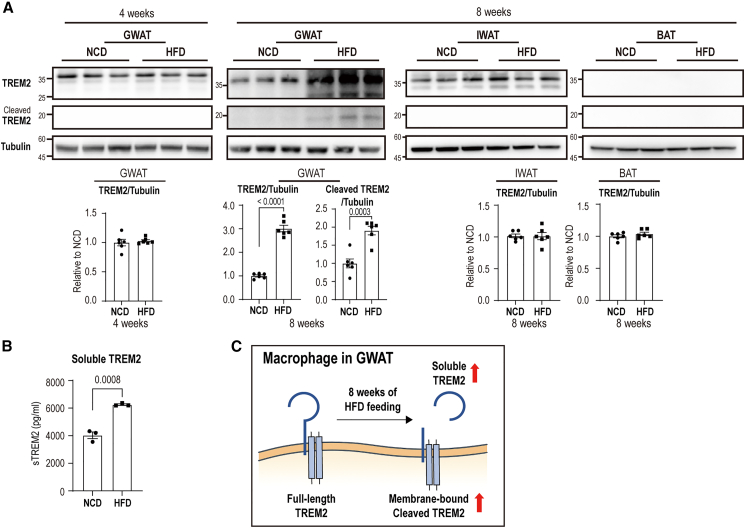
High-fat diet feeding increases cleaved TREM2 and TREM2 levels in gonadal white adipose tissue (A) Immunoblot analysis of TREM2 and cleaved TREM2 levels in gonadal white adipose tissue (GWAT), inguinal white adipose tissue (IWAT), and brown adipose tissue (BAT) of mice fed a high-fat diet (HFD) and normal chow diet (NCD) for 4 and 8 weeks (*n* = 6 mice per group). (B) ELISA-based quantification of soluble TREM2 (sTREM2) levels in mouse serum (*n* = 3 mice per group). (C) Schematic diagram illustrating cleaved membrane-bound TREM2 and soluble TREM2 forms. Data are presented as mean values ± SEM. *p* values were determined by the unpaired two-sided Student’s *t* test and were annotated directly in the figure at the corresponding comparisons.

To determine whether HFD feeding induces TREM2 cleavage in WAT, we performed immunoblot analyses on GWAT from mice fed an HFD for 8 weeks. The results showed approximately a 3-fold increase in full-length and 2-fold increase in its cleaved form, indicating that HFD feeding promotes both TREM2 expression and proteolytic shedding in GWAT (Figure 1A). Increased expression of both full-length and cleaved TREM2 was not observed in the GWAT of mice fed on HFD for 4 weeks (Figure 1A), suggesting that the upregulation and cleavage of TREM2 occur as a consequence of more chronic HFD exposure. Consistent with elevated TREM2 cleavage observed in GWAT, serum levels of sTREM2 were significantly elevated in mice after 8 weeks of HFD feeding (Figures 1B and 1C). In contrast to GWAT, TREM2 expressions remained unchanged in brown adipose tissue (BAT) and inguinal white adipose tissue,^25^ following 8 weeks of HFD feeding (Figure 1A). Collectively, these findings indicate that HFD feeding for 8 weeks selectively induces TREM2 expression and cleavage in GWAT.

### Obesity enhances ADAM10/17 expression in adipose tissue macrophages in both humans and mice

**Figure 2:**
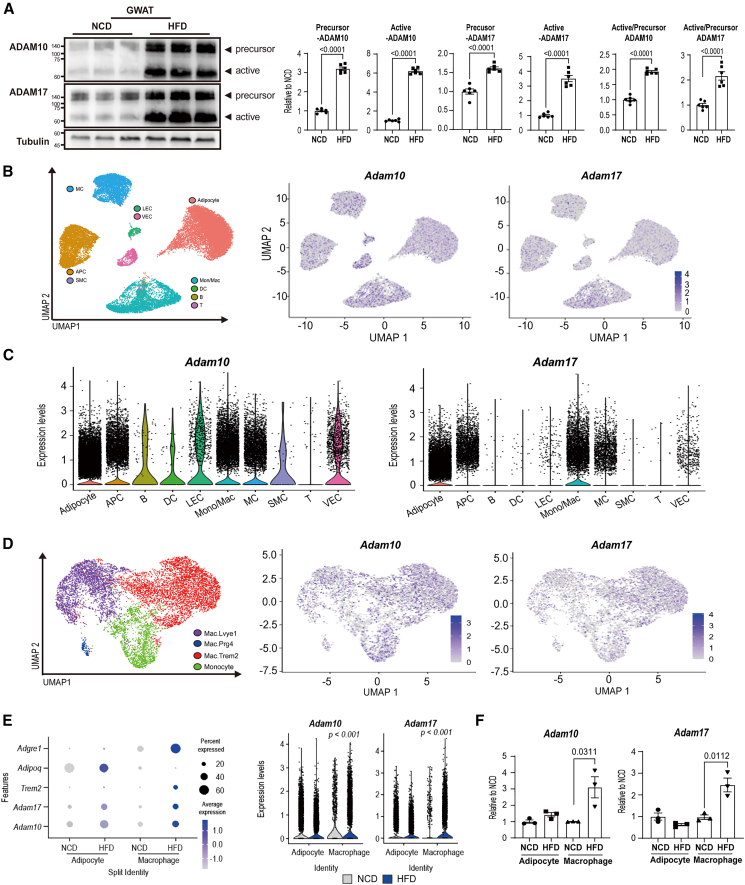
HFD feeding upregulates *Adam10* and *Adam17* expression in monocyte/macrophage populations of GWAT (A) Immunoblot analysis of ADAM10 and ADAM17 protein expression in gonadal white adipose tissue (GWAT) of mice after 8 weeks of NCD or HFD feeding (*n* = 6 mice per group). (B) Umap plot of *Adam10*, *Adam17*, and total nuclei isolated from GWAT of NCD and HFD-fed mice (PRJNA942977). Clusters are colored by cell types: adipocyte, mesothelial cell, lymphatic endothelial cell, vascular endothelial cell, adipocyte progenitor cell, smooth muscle cell (SMC), monocyte/macrophage (mono/mac), dendritic cell (DC), B cell, and T cell. (C) Violin plot of *Adam10* and *Adam17* gene expression levels in total cell types from GWAT of NCD- and HFD-fed mice. (D) Umap plot of *Adam10*, *Adam17* from monocyte/macrophage population in GWAT of NCD- and HFD-fed mice (PRJNA942977). Clusters are colored by cell types: *Lyve1*+macrophage (Mac.*Lyve1*), *Trem2*+macrophage (Mac.*Trem2*), *Prg4*+macrophage (Mac.*Prg4*), and monocyte. (E) Bubble plot and violin plot of *Adam10* and *Adam17* expression levels in distinct cell populations from mouse GWAT, determined by single-nucleus RNA sequencing (PRJNA942977). (F) qPCR analysis of *Adam10* and *Adam17* mRNA expression in isolated F4/80+ macrophages and adipocytes from GWAT after 8 weeks of NCD or HFD feeding (*n* = 3 mice per group). Data are presented as mean values ± SEM. *p* values were determined by the unpaired two-sided Student’s *t* test and were annotated directly in the figure at the corresponding comparisons.

In AD, ectodomain shedding of TREM2 is enhanced by ADAM10/17 proteases, and sTREM2 regulates neuroinflammatory responses.^14^ Thus, we investigated whether HFD feeding increased ADAM10/17 expression levels in GWAT. After 8 weeks of HFD feeding, both the active and precursor forms of ADAM10 and ADAM17 were markedly elevated in GWAT, along with a significant elevation in the active-to-precursor ratios (Figure 2A). Consistent with these findings, publicly available bulk RNA sequencing datasets (GSE86080, GSE280875: GWAT of normal chow diet [NCD] and HFD-fed mice for 12 weeks, and GSE182930: GWAT of NCD and HFD fed mice for 8 weeks) demonstrated that *Adam10* and *Adam17* expression were dramatically increased in GWAT of 8 or 12 weeks of HFD-fed mice (Figure S1). Our single-nucleus RNA sequencing dataset (snRNAseq; PRJNA942977) revealed that *Adam17* was highly expressed in monocyte/macrophage populations in GWAT, whereas *Adam10* was expressed in various cell types (Figures 2B and 2C). Specifically, *Adam10* and *Adam17* were enriched in TREM2-positive macrophages within GWAT (Figure 2D), and their expressions were significantly upregulated by HFD feeding in monocyte/macrophage populations compared to adipocytes (Figure 2E). To validate these transcriptomic data, we isolated F4/80+ cells and floating adipocytes from GWAT by magnetic activated cell sorting (MACS), and we observed that *Adam10* and *Adam17* expression levels were selectively elevated in F4/80^+^ macrophages in GWAT (Figure 2F).

**Figure 3:**
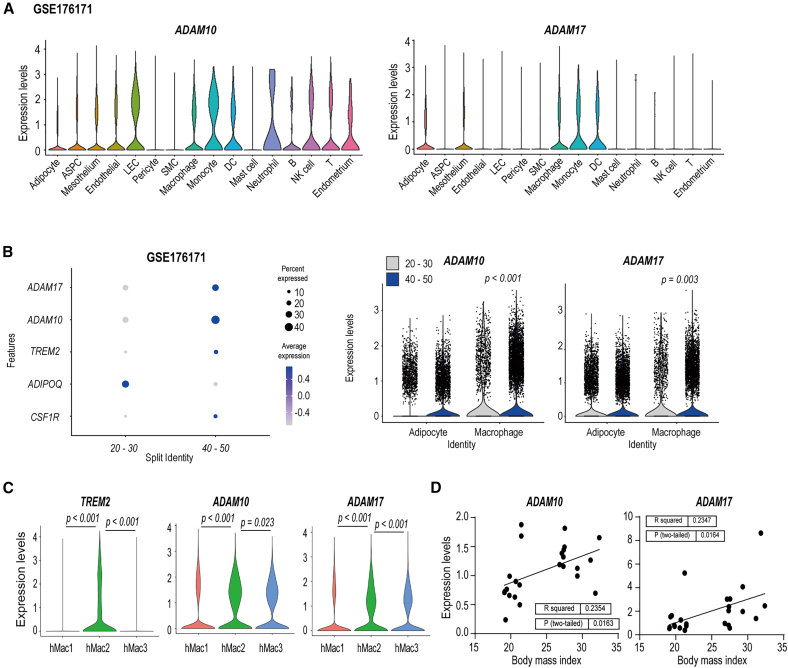
Obesity increases *ADAM10* and *ADAM17* expression in macrophage populations of WAT (A) *ADAM10* and *ADAM17* expression levels in total cell types of human visceral adipose tissues (GSE176171). Clusters are colored by cell types: Adipocyte, adipose stem and progenitor cells (ASPC), mesothelium, endothelial cell, lymphatic endothelial cell (LEC), pericyte, smooth muscle cell (SMC), macrophage, monocyte, dendritic cell (DC), mast cell, neutrophil, B cell, natural killer cell (NK cell), T cell, and endometrium cell. (B) Bubble plot and violin plot of *ADAM10* and *ADAM17* gene expressions and representative markers in adipocyte and macrophage populations with body mass index (BMI) in human subcutaneous adipose tissue (GSE176171). (C) Violin plot of *TREM2*, *ADAM10*, and *ADAM17* gene expression levels in human macrophage sub-populations (hMac1, hMac2, hMac3). (D) Correlation analysis of *ADAM10* and *ADAM17* gene expression levels with BMI in human subcutaneous adipose tissue (*n* = 12 patients per group). Data are presented as mean values ± SEM. *p* values were determined by the unpaired two-sided Student’s *t* test and were annotated directly in the figure at the corresponding comparisons.

Publicly available human single cell/nucleus RNA sequencing dataset (GSE176171)^26^ also demonstrated that *ADAM17* expression was highly enriched in macrophages and monocytes, whereas *ADAM10* was broadly expressed across multiple cell types (Figure 3A). Both genes were significantly upregulated in macrophages compared to adipocytes with increasing body mass index (BMI) (Figure 3B). Violin plots further demonstrated that *ADAM10* and *ADAM17* were selectively enriched in hMac2 clusters, which selectively express *TREM2* (Figure 3C). Consistent with scRNAseq analysis, we validated *ADAM10* and *ADAM17* expression levels were positively correlated with BMI in human subcutaneous WAT (Figure 3D). Taken together, these results indicated that HFD feeding significantly increased both precursor and active ADAM10/17 within GWAT, predominantly in macrophages, and that their expressions were positively correlated with BMI in humans.

### Co-culture with pyroptotic, but not apoptotic, adipocytes induces ADAM10/17 activation and TREM2 cleavage in RAW264.7 cells

**Figure 4:**
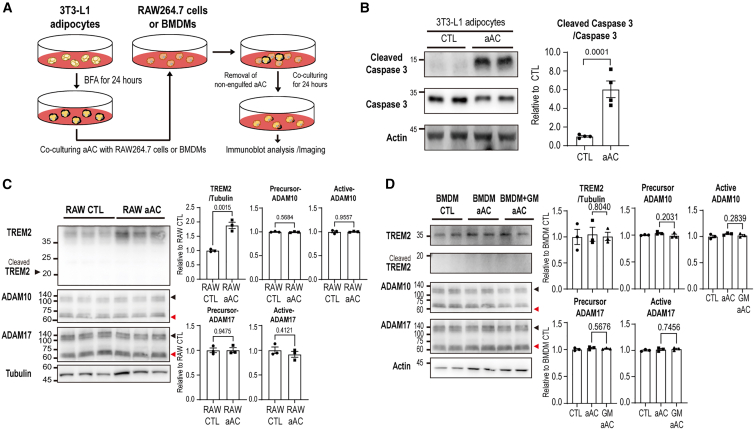
Apoptotic adipocytes fail to induce TREM2 shedding in RAW264.7 cells and bone marrow-derived macrophages (BMDMs) (A) Schematic diagram illustrating the experimental method used for co-culturing dying/dead adipocytes with RAW264.7 cells or BMDMs. Apoptotic adipocytes (aAC) were generated by treating differentiated 3T3-L1 adipocytes with brefeldin A (BFA; 5 μg/mL for 24 h). Apoptotic adipocytes (1 × 10^5^ cells/well) were directly co-cultured with RAW264.7 cells or BMDMs (5 × 10^5^ cells/well) in growth medium. After 24 h of co-culture, non-engulfed or floating apoptotic adipocytes were removed by PBS washing, and RAW264.7 cells or BMDMs were subsequently harvested for immunoblot analysis. (B) Immunoblot analysis of caspase-3 expression levels in 3T3-L1 adipocytes after BFA for 24 h (*n* = 3 cells per group). (C) and (D) Immunoblot analysis of TREM2, ADAM10, and ADAM17 protein expression levels in RAW264.7 cells (C) and BMDMs (D) co-cultured with apoptotic adipocytes (*n* = 3 cells per group). Black arrow and red arrow indicate each precursor form of ADAM10/17 and active form of ADAM10/17. Data are presented as mean values ± SEM. *p* values were determined by the unpaired two-sided Student’s *t* test and were annotated directly in the figure at the corresponding comparisons.

Previous studies have shown that TREM2 plays a critical role in signal transduction associated with the clearance of dying cells.^25^ Given that TREM2 is cleaved between His^157^ and Ser^158^ by ADAM10 and ADAM17, we hypothesized that increased TREM2 shedding may impair phagocytic activity. To investigate the specific cellular cues that promote TREM2 shedding in macrophages, we first examined whether apoptotic adipocytes influence the activation of ADAM10/17 and the cleavage of TREM2 (Figure 4A). Apoptosis was induced by treating 3T3-L1 adipocytes with brefeldin A (BFA) for 24 h, and successful induction was confirmed by immunoblotting for cleaved caspase-3. (Figure 4B). RAW264.7 cells were co-cultured with apoptotic adipocytes for 24 h, but this condition failed to induce detectable activation of ADAM10 or ADAM17, nor did it lead to the appearance of cleaved TREM2 (Figure 4C). Notably, however, the overall expression of TREM2 was modestly increased under these conditions (Figure 4C), suggesting that while apoptotic cells may influence TREM2 transcriptionally or translationally, they do not trigger its proteolytic processing. We further confirmed these findings in mouse bone marrow-derived macrophages (BMDMs), where co-culture with apoptotic adipocytes also failed to induce ADAM10/17 activation or TREM2 cleavage (Figure 4D).

**Figure 5:**
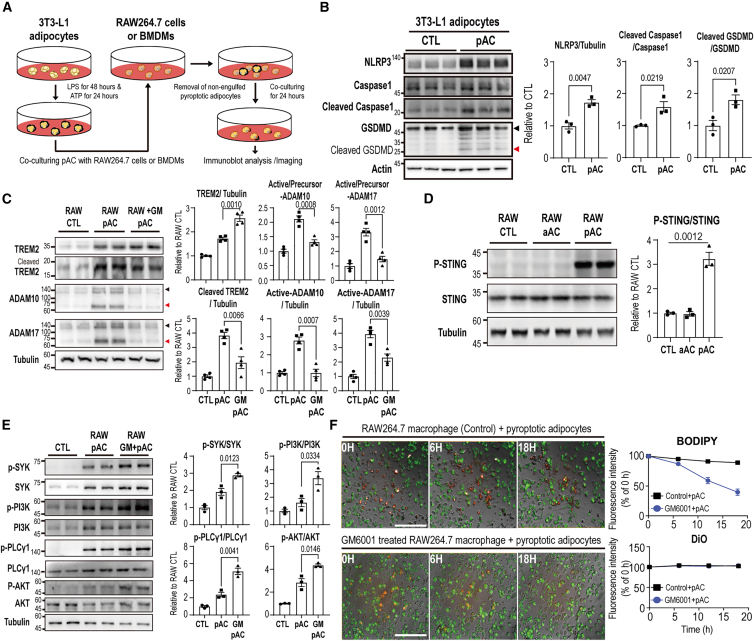
GM6001 blocks TREM2 shedding in RAW264.7 cells induced by pyroptotic adipocytes (A) A schematic diagram illustrating the experimental method used for co-culturing pyroptotic adipocytes with macrophages. Pyroptotic adipocytes were generated by treating differentiated 3T3L1 adipocytes with lipopolysaccharide (LPS; 100 μg/μL, 48 h) followed by ATP (2 mM, 24 h). Pyroptotic adipocytes (1 × 10^5^ cells/well) were directly co-cultured with RAW264.7 cells (5 × 10^5^ cells/well) or BMDMs for 24 h in growth medium. After co-culture, non-engulfed or floating pyroptotic adipocytes were removed by PBS washing, and RAW264.7 cells and BMDMs were subsequently harvested for immunoblot analysis. (B) Immunoblot analysis of NLRP3, caspase-1, and GSDMD (gasdermin D) expression levels in 3T3-L1 adipocytes after LPS priming for 48 h and ATP treatment for 24 h (*n* = 3 cells per group). Black arrow and red arrow indicate each total form of GSDMD and cleaved GSDMD. (C) Immunoblot analysis of TREM2, ADAM10, and ADAM17 expression levels in RAW264.7 cells co-cultured with pyroptotic adipocytes for 24 h in the presence or absence of GM6001 (*n* = 4 cells per group). Black arrow and red arrow indicate each precursor form of ADAM10/17 and active form of ADAM10/17. (D) Immunoblot analysis of P-STING and STING expression levels in RAW264.7 cells co-cultured with pyroptotic adipocytes (*n* = 3 cells per group). (E) Immunoblot analysis of P-syk, syk, P-PI3K, PI3K, P-PLCγ1, PLCγ1, P-AKT, and AKT protein levels in RAW264.7 cells co-cultured with pyroptotic adipocytes (*n* = 3 cells per group). (F) Phagocytosis analysis of RAW264.7 cells co-cultured with pyroptotic adipocytes for 18 h in the presence of GM6001. Adipocytes were tagged with C12-BODIPY (red), and macrophages were stained with DiO (green). Representative images from three independent experiments are shown, with quantification provided in the right panel. Scale bars, 100 μm. Data are presented as mean values ± SEM. *p* values were determined by the unpaired two-sided Student’s *t* test and were annotated directly in the figure at the corresponding comparisons.

Pyroptosis, in which gasdermin D (GSDMD) forms membrane pores and drives the release of interleukin-1β, is known to activate metalloproteinases such as ADAM17.^27^^,^^28^ To test whether pyroptotic adipocytes can promote TREM2 cleavage via this mechanism, we co-cultured RAW264.7 cells with pyroptotic adipocytes for 24 h (Figure 5A). Pyroptotic adipocytes induced by lipopolysaccharide (LPS) and ATP, and its successful induction was validated by increased nucleotide-binding domain, leucine-rich repeat, and pyrin domain-containing protein 3 (NLRP3), cleaved caspase-1 and cleaved GSDMD expression levels (Figure 5B). This condition led to a robust activation of both ADAM10 and ADAM17, as well as a marked increase in cleaved TREM2 levels in RAW264.7 cells (Figure 5C).

Previous studies demonstrated that stimulator of interferon genes (STING) phosphorylation promotes activation of ADAM17 in RAW264.7 cells.^29^ Furthermore, the STING pathway plays a critical role in detecting damage-associated molecular patterns (DAMPs) derived from damaged cells and contributes to the initiation of innate immune responses.^30^ Based on these findings, we hypothesized that co-culturing RAW264.7 cells with pyroptotic adipocytes induces activation of the STING pathway and subsequently upregulates ADAM17. Co-culture of RAW264.7 cells with pyroptotic adipocytes led to a marked increase in STING phosphorylation, whereas apoptotic adipocytes failed to increase phosphorylation of STING (Figure 5D). These observations suggest that pyroptotic, but not apoptotic, adipocytes trigger STING phosphorylation in macrophages, potentially contributing to the upregulation of ADAM proteases and TREM2 cleavages.

### GM6001 treatment promotes the clearance of pyroptotic adipocytes by RAW264.7 cells, BMDMs, and THP-1 cells

**Figure 6:**
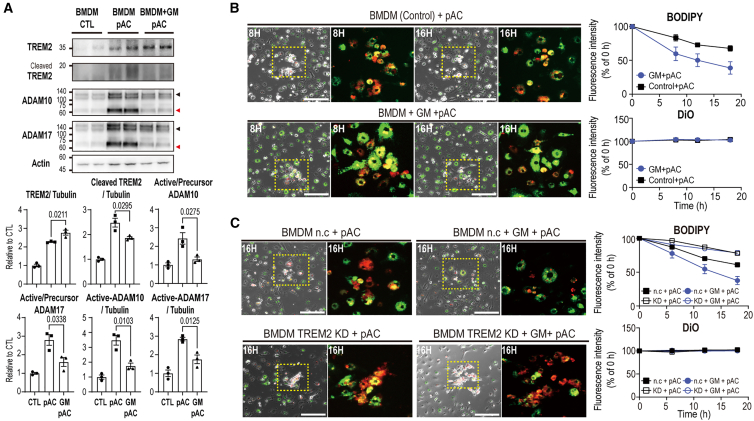
GM6001 prevents the cleavage of TREM2 on BMDMs induced by pyroptotic adipocytes (A) Immunoblot analysis of TREM2, ADAM10, and ADAM17 expression levels in BMDMs co-cultured with pyroptotic adipocytes for 24 h in the presence or absence of GM6001 (*n* = 3 cells per group). Black arrow and red arrow indicate each precursor form of ADAM10/17 and active form of ADAM10/17. (B) and (C) Phagocytosis analysis of BMDMs, (B) TREM2 knockdown^32^ BMDMs, and negative control (NC). (C) co-cultured with pyroptotic adipocytes for 18 h in the presence of GM6001. Adipocytes were tagged with C12-BODIPY (red), and macrophages were stained with DiO (green). Representative images from three independent experiments are shown, with quantification provided in the right panel. The yellow boxes indicate the regions shown in the magnified images. Scale bars, 200 μm. Data are presented as mean values ± SEM. *p* values were determined by the unpaired two-sided Student’s *t* test and were annotated directly in the figure at the corresponding comparisons.

Data suggest that inflammatory signals derived from pyroptotic adipocytes impair the functional capacity of macrophages to clear dying cells, possibly through excessive TREM2 cleavage. To evaluate whether inhibition of ADAM10/17 activity could mitigate TREM2 shedding and restore phagocytosis, we treated RAW264.7 cells with GM6001, a broad-spectrum MMP/ADAM protease inhibitor, prior to co-culturing with adipocytes. GM6001 treatment attenuated the levels of active ADAM10 and ADAM17 and decreased TREM2 cleavage, leading to increased expression of full-length TREM2 (Figure 5C). TREM2 is known to facilitate phagocytosis through activation of the spleen tyrosine kinase (SYK)/phosphatidylinositol 3-kinase (PI3K)/phospholipase C gamma 1 (PLCγ1)/protein kinase B (AKT) signaling cascade in macrophages.^31^ We demonstrated that GM6001 treatment enhanced the phosphorylation of SYK/PI3K/PLCγ1/AKT in RAW264.7 cells exposed to pyroptotic adipocytes (Figure 5E). Consistently, GM6001 improved the phagocytic capacity of RAW264.7 cells co-cultured with pyroptotic adipocytes (Figure 5F). To validate these findings in a primary cell model, we isolated BMDMs from mice and repeated phagocytosis assays. Consistent with the results in RAW264.7 cells, GM6001 treatment inhibited ADAM10/17 activation and TREM2 shedding (Figure 6A), while enhancing phagocytosis of adipocytes in BMDMs (Figure 6B). Furthermore, in THP-1 cells, GM6001 treatment also increased phagocytic activity toward pyroptotic adipocytes (Figure S2).

Next, to determine whether the GM6001-mediated enhancement of pyroptotic adipocyte efferocytosis is dependent on TREM2, we performed co-culture experiments using TREM2 knockdown (KD) BMDM.^32^ Immunoblot analysis validated that TREM2 protein expression was reduced by more than 43.8% in KD BMDM (Figure S3). The co-culture experiment displayed that GM6001 treatment failed to enhance the phagocytic activity of TREM2 KD BMDMs toward pyroptotic adipocytes (Figure 6C). Together, inhibition of ADAM10/17 activity by GM6001 restored TREM2-mediated phagocytic clearance of pyroptotic adipocytes by RAW264.7 cells.

### GM6001 attenuates HFD-induced TREM2 shedding and metabolic dysfunction

**Figure 7:**
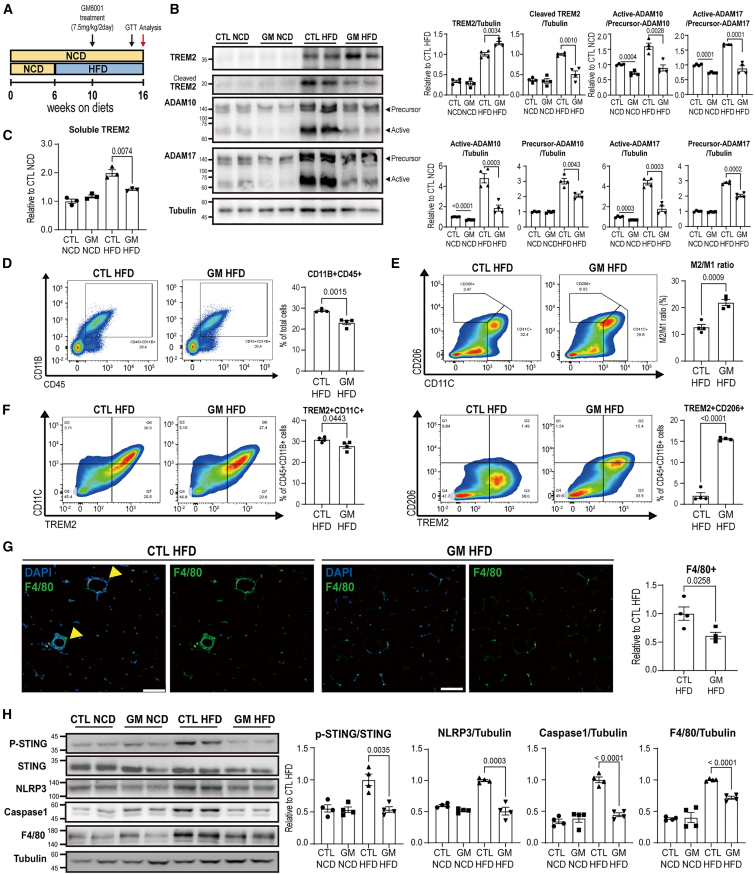
GM6001 treatment reduces TREM2 shedding and HFD-induced inflammation in GWAT (A) Schematic illustration of the experimental strategy for GM6001 administration (7.5 mg/kg/2 days) in mice fed a high-fat diet (HFD) for 10 weeks. (B) Immunoblot analysis of TREM2, ADAM10, and ADAM17 protein levels in gonadal white adipose tissue (GWAT) of HFD-fed mice treated with or without GM6001 (*n* = 4 mice per group). (C) Measurement of soluble TREM2 (sTREM2) levels in the serum of NCD- or HFD-fed mice treated with GM6001 for 10 weeks (*n* = 3 mice per group). (D–F) Representative flow profile and quantification of (D) CD11B + CD45^+^ cells (E) M2/M1 macrophage ratio (CD206^+^CD11C^−^CD45^+^CD11B^+^/CD11C^+^CD206^−^CD45^+^CD11B^+^), and (F) TREM2^+^CD11C^+^ and TREM2^+^CD206^+^ macrophage populations in GWAT of GM6001-treated mice after feeding HFD for 10 weeks (*n* = 4 mice per group). (G) Immunofluorescence staining of F4/80 (green) with DAPI (blue) counterstaining in paraffin-embedded GWAT sections from GM6001-treated and control mice (*n* = 4 mice per group, scale bars, 100 μm). (H) Immunoblot analysis of P-STING, STING, NLRP3, F4/80, and caspase-1 expression levels in GWAT of HFD-fed mice treated with or without GM6001 (*n* = 4 mice per group). Data are presented as mean values ± SEM. *p* values were determined by the unpaired two-sided Student’s *t* test and were annotated directly in the figure at the corresponding comparisons.

To investigate whether inhibiting ADAM10/17-mediated TREM2 cleavage could alleviate HFD-induced metabolic dysfunction, we treated GM6001 to HFD-fed mice. GM6001 was injected at 2-day intervals beginning at 4 weeks of HFD feeding, prior to the onset of increased TREM2 expression and cleavage (Figures 1A and 7A). Immunoblot analysis revealed that GM6001 treatment effectively reduced cleaved TREM2 levels, while increasing total TREM2 in GWAT (Figure 7B). In parallel, both active and precursor forms of ADAM10 and ADAM17, as well as their active to precursor ratios, were markedly decreased by GM6001 (Figure 7B). Consistent with immunoblot analysis, GM6001 reduced the elevated sTREM2 levels by HFD feeding (Figure 7C).

Since HFD feeding stimulates pro-inflammatory polarization of adipose tissue macrophages and decreases anti-inflammatory-related genes in GWAT,^3^ we next examined whether GM6001 modulates immune cell phenotypes in GWAT of HFD-fed mice. Flow cytometry analysis showed that GM6001 led to a reduction in the total macrophage population (CD11B + CD45^+^) within GWAT (Figure 7D). Notably, GM6001 treatment not only significantly increased M2/M1 ratio (M2-like macrophages [CD45^+^CD11B + CD11C-CD206+] over M1-like macrophages [CD45^+^CD11B + CD206-CD11C+]) in GWAT (Figure 7E), but also reshaped the characteristic of TREM2 positive macrophages, promoting a phenotypic shift from pro-inflammatory related TREM2+CD11C+ macrophages toward anti-inflammatory related TREM2+CD206+ macrophages (Figure 7F). In line with these observations, we also detected the reduction in macrophage recruitments with decreased F4/80 expression levels following GM6001 treatment (Figures 7G and 7H) and the elevated expression of anti-inflammatory genes (*Cd163*, *Arg1*, and *Il-10*; Figure S4). Considering GM6001 treatment promoted M2-like macrophage polarization and increased anti-inflammatory gene expression in GWAT, we speculated that this effect might involve suppression of DAMP releases and inhibition of STING signaling, a known upstream regulator of ADAM17.^29^ Immunoblot analysis showed the reduced levels of phosphorylated STING in GM6001-treated HFD-fed mice (Figure 7H). In addition, GM6001 treatment decreased the expression of NLRP3 and caspase-1 in GWAT, suggesting reduced activation of programmed cell death and the release of DAMPs (Figure 7H).

**Figure 8:**
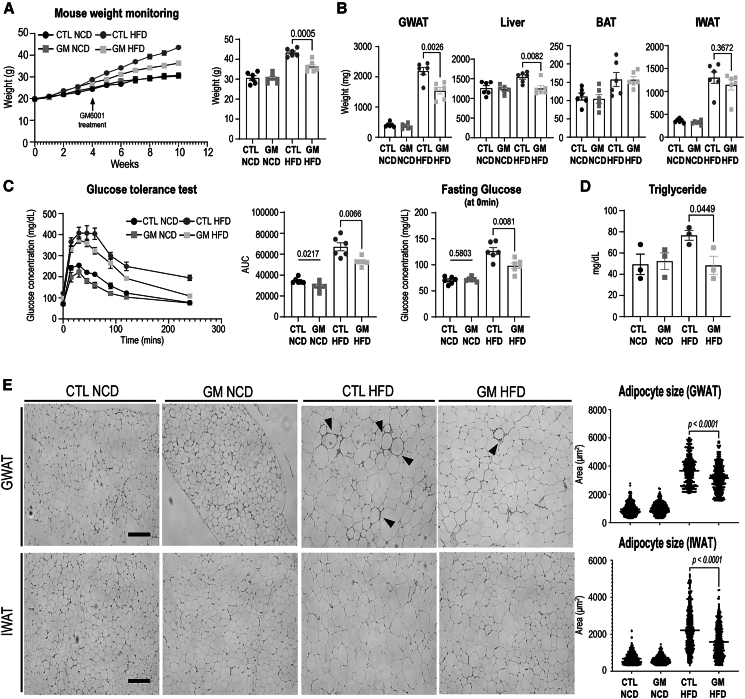
GM6001 treatment reduces HFD-induced metabolic dysfunction (A) Body weight monitoring of mice fed a normal chow diet (NCD) or high-fat diet (HFD) with or without GM6001 (7.5 mg/kg/2 days) treatment for 10 weeks (*n* = 6 mice per group). (B) Weight measurements of adipose tissues and liver from NCD- or HFD-fed mice following GM6001 treatment (*n* = 6 mice per group). (C) Intraperitoneal glucose tolerance test performed at 8 weeks of HFD feeding in NCD- or HFD-fed mice treated with GM6001 (*n* = 6 mice per group). (D) Quantification of serum triglyceride levels in mice fed NCD or HFD for 10 weeks with or without GM6001 treatment (*n* = 4 mice per group). (E) Representative H&E images and quantification of adipocyte size in GWAT sections from NCD- or HFD-fed mice treated with GM6001 for 10 weeks (scale bars, 100 μm). Data are presented as mean values ± SEM. *p* values were determined by the unpaired two-sided Student’s *t* test and were annotated directly in the figure at the corresponding comparisons.

Next, we also assessed whether GM6001 treatment could ameliorate HFD-induced metabolic dysfunction. After 6 weeks of treatment, GM6001 significantly reduced mouse weights, inguinal white adipose tissue (IWAT), and GWAT mass in HFD-fed mice, whereas no notable changes were observed in NCD-fed controls (Figures 8A and 8B). Additionally, GM6001 restored HFD-induced glucose intolerance and hyperinsulinemia (serum triglyceride) in HFD-fed mice, suggesting a partial rescue of HFD-induced metabolic impairments (Figures 8C and 8D). Adipocyte area analysis in GWAT and IWAT revealed that GM6001 treatment restricted HFD-induced adipocyte hypertrophy (Figure 8E). Furthermore, as the decrease of F4/80+ macrophage recruitments (Figure 7G), the crown-like structures (black arrows), which macrophage clusters surrounding dead adipocytes, were observed less frequently in GM6001-treated GWAT of HFD-fed mice (Figure 8E). Together, these findings suggest that pharmacological inhibition of ADAM10/17 by GM6001 may attenuate TREM2 cleavage and contribute to the mitigation of metabolic imbalance under HFD conditions.

## Discussion

Obesity is characterized by chronic low-grade inflammation in adipose tissue, accompanied by the recruitment and functional reprogramming of tissue-resident and infiltrating immune cells.^33^ Among these, LAMs, marked by the expression of TREM2, have emerged as key regulators of lipid metabolism and tissue remodeling in obese adipose depots.^5^ However, the precise role of TREM2 in adipose tissue homeostasis remains unclear, with conflicting reports suggesting both protective and deleterious effects in the context of obesity.^5^^,^^34^ These paradoxical findings highlight a critical knowledge gap in our understanding of TREM2^+^ macrophage function under metabolic stress. We hypothesized that while TREM2^+^ macrophage recruitment may initially serve a compensatory role in clearing lipid debris and dying adipocytes, inflammatory cues within the obese adipose microenvironment may drive pathological TREM2 cleavage, thereby impairing macrophage function. In this study, we aimed to elucidate the molecular mechanisms underlying TREM2 dysfunction in obesity, with a particular focus on the role of pyroptotic adipocyte death and ADAM-mediated TREM2 shedding.

In the current study, we identified a novel mechanism by which pyroptotic adipocyte death contributes to macrophage dysfunction in obese adipose tissue. Specifically, we found that DAMPs released from pyroptotic adipocytes activate STING signaling in macrophages, leading to the upregulation of ADAM10 and ADAM17. This, in turn, promotes the proteolytic cleavage of TREM2, resulting in a loss of surface-bound TREM2 and impaired phagocytic function. Therefore, TREM2^+^ macrophages lose their capacity to efficiently clear dying adipocytes and resolve inflammation, contributing to chronic adipose tissue inflammation during HFD-induced obesity. These findings uncover a previously unrecognized link between pyroptotic adipocyte death and pathological TREM2 processing in adipose tissue.

While the role of TREM2 has been well characterized in the central nervous system, where excessive ADAM10/17-mediated TREM2 shedding exacerbates neuro-inflammation and impairs microglial function,^35^ its role in peripheral tissues has remained less clear. Recent studies have shown that increased TREM2 shedding and sTREM2 levels are associated with hepatic lipid accumulation, MASH, and chronic liver disease.^7^^,^^23^ Our findings extend these observations to adipose tissue, demonstrating that ADAM10/17-driven TREM2 cleavage similarly disrupts macrophage function in the context of obesity. By impairing lipid handling and clearance of dying adipocytes, pathological TREM2 shedding may amplify inflammation and contribute to the metabolic derangements observed in obese adipose tissue.

We further demonstrated that TREM2 cleavage in response to HFD feeding occurs in a depot-specific manner, with significant accumulation of both full-length and cleaved TREM2 observed in GWAT, but not in BAT and IWAT. This was accompanied by a marked upregulation of ADAM10 and ADAM17 in GWAT macrophages. Pharmacological inhibition of these proteases using GM6001 not only suppressed TREM2 cleavage but also led to notable metabolic improvements, including reduced weight gain, smaller adipocyte size, lower serum triglyceride levels, and improved glucose tolerance. Our *in vitro* findings further revealed that pyroptotic, but not apoptotic, adipocytes trigger STING-dependent activation of ADAM10/17 and subsequent TREM2 shedding in macrophages. GM6001 treatment effectively preserved full-length TREM2 and restored phagocytic function under these inflammatory conditions, suggesting that excessive TREM2 shedding compromises the macrophage-mediated clearance of dying adipocytes. Notably, GM6001 treatment not only significantly elevated the M2/M1 macrophage ratio in GWAT but also induced a marked phenotypic reprogramming of TREM2^+^ macrophages. Specifically, GM6001 shifted the macrophage population from a pro-inflammatory TREM2^+^CD11C^+^ phenotype toward an anti-inflammatory TREM2^+^CD206^+^ phenotype, highlighting its potential role in modulating macrophage functional plasticity within adipose tissue.

### Limitations of the study

Although GM6001 improved macrophage phagocytosis and systemic metabolism, it is a broad-spectrum metalloprotease inhibitor and may exert off-target effects. ADAM10/17 are pleiotropic sheddases with numerous substrates beyond TREM2.^36^^,^^37^^,^^38^ Therefore, the observed benefits cannot be attributed exclusively to inhibition of TREM2 shedding. While our results from TREM2 KD macrophages demonstrate that the pro-phagocytic effect of GM6001 is TREM2-dependent, we cannot fully exclude contributions from other ADAM10/17 substrates. To further elucidate the mechanisms by which TREM2 cleavage contributes to adipose tissue inflammation, future studies using adeno-associated virus (AAV)-mediated TREM2 overexpression or sheddase-resistant mutant TREM2 knockin mouse models will be required. Additionally, while our findings provide mechanistic insight into the regulation of TREM2 cleavage in murine models, further validation in human adipose tissue will be essential to establish its relevance in human obesity and metabolic disease.

Another limitation of our study is the inability to definitively identify the tissue sources of circulating sTREM2. While our data suggest adipose tissue macrophages may contribute to sTREM2 production under HFD feeding, other tissues cannot be excluded. Previous studies have shown that prolonged western diet (containing high-fat, sucrose, and cholesterol) feeding induces TREM2 expression in the liver, including in Kupffer cells and other liver-resident macrophages,^7^ suggesting a potential hepatic contribution. Moreover, since TREM2 is also expressed in the brain, central nervous system–derived sTREM2 may additionally account for the elevated circulating levels observed in obesity.

In summary, our findings identify proteolytic cleavage of TREM2 as a key regulatory event driving macrophage dysfunction in adipose tissue during HFD-induced obesity. We highlight pyroptotic adipocyte death as an important mode of cell death that promotes pathological tissue remodeling by inducing STING-dependent activation of ADAM10/17 and subsequent TREM2 shedding. This process reprograms macrophages toward a dysfunctional, pro-inflammatory state, impairing their ability to clear dying adipocytes and resolve inflammation. Targeting this pathway by limiting TREM2 shedding through ADAM10/17 inhibition may represent a promising therapeutic approach for mitigating adipose tissue inflammation and systemic metabolic dysfunction associated with obesity.

## Resource availability

### Lead contact

Requests for additional information or resources should be directed to the lead contact, Yun-Hee Lee (yunhee.lee@snu.ac.kr).

### Materials availability

This study did not generate new unique reagents.

### Data and code availability

• SnRNA sequencing data are available via NCBI Sequence Read Archive (SRA): PRJNA1231064 (https://www.ncbi.nlm.nih.gov/bioproject/PRJNA942977) and NCBI Gene Expression Omnibus (GEO): GSE176171 (https://www.ncbi.nlm.nih.gov/geo/query/acc.cgi?acc=GSE176171) are publicly available. All RNA sequencing data are available via their respective NCBI GEO databases under accession codes GSE86080 (https://www.ncbi.nlm.nih.gov/geo/query/acc.cgi?acc=GSE86080), GSE182930 (https://www.ncbi.nlm.nih.gov/geo/query/acc.cgi?acc=GSE182930), and GSE280875 (https://www.ncbi.nlm.nih.gov/geo/query/acc.cgi?acc=GSE280875).
• This article does not report original computer code, algorithms, or computational models.
• Original western blot has been deposited at Mendeley data and are publicly available at https://doi.org/10.17632/ds6smymmx2.1. Any additional information required to reanalyze the data reported in this article is available from the lead contact upon request.

## Acknowledgments

This research was supported by the 10.13039/501100003725National Research Foundation of Korea (NRF) grants (RS-2023-00213572, RS-2024-00400118, and RS-2025-16063709) funded by the 10.13039/501100014188Ministry of Science and ICT (MSIT) of the Korean Government; by 10.13039/501100003661Korea Institute for Advancement of Technology (KIAT) grant funded by the Korea Government (10.13039/501100003052MOTIE) (RS-2025-02214034, HRD Program for Industrial Innovation); and by 10.13039/100016341Korea University Guro Hospital grant (Korea research-driven hospital) funded by 10.13039/100016989Korea University Medicine (no. K2210441).

## Author contributions

Y.-H.L. conceived and designed the study. C.C., J.L., and G.P. conducted the animal experiments. C.C. performed *in vitro* experiments and analyzed human samples. S.N. provided human samples. Y.-H.L. and C.C. wrote the manuscript. All authors reviewed the manuscript.

## Declaration of interests

The authors declare no competing interests**.**

## STAR★Methods

### Key resources table

**Table:** 

REAGENT or RESOURCE	SOURCE	IDENTIFIER
**Antibodies**		

α/β-Tubulin Rabbit mAb	Cell Signaling Technology	Cat# 2148; RRID:AB_2288042
Β-Actin (C-4) Mouse mAb	Santa Cruz Biotechnology	Cat# sc-47778; RRID:AB_626632
TREM2 (E7P8J) Rabbit mAb (Carboxy-terminal Antigen)	Cell Signaling Technology	Cat# 76765; RRID:AB_2799888
TREM2 (D8I4C) Rabbit mAb	Cell Signaling Technology	Cat# 91068; RRID:AB_2721119
Syk (D3Z1E) XP® Rabbit mAb	Cell Signaling Technology	Cat# 13198; RRID:AB_2687924
Phospho-Syk (Tyr525/526) (C87C1) Rabbit mAb	Cell Signaling Technology	Cat# 2710; RRID:AB_2197222
PLCγ1 (D9H10) XP® Rabbit mAb	Cell Signaling Technology	Cat# 5690; RRID:AB_10691383
Phospho-PLCγ1 (Tyr783) (D6M9S) Rabbit mAb	Cell Signaling Technology	Cat# 14008; RRID:AB_2728690
ADAM10 (B-3) Mouse mAb	Santa Cruz Biotechnology	Cat# sc-28358; RRID:AB_626636
ADAM17/TACE Rabbit mAb	Cell Signaling Technology	Cat# 3976; RRID:AB_2242380
Phospho-STING (Ser365) (D8F4W) Rabbit mAb	Cell Signaling Technology	Cat# 72971; RRID:AB_2799831
STING (D2P2F) Rabbit mAb	Cell Signaling Technology	Cat# 13647; RRID:AB_2732796
anti-NLRP3/NALP3 (Cryo-2) Mouse mAb	AdipoGen	Cat# AG-20B-0014-C100; RRID:AB_2490202
anti-Caspase-1 (p20) Mouse mAb (Casper-1)	AdipoGen	Cat# AG-20B-0042; RRID:AB_2490248
F4/80 (D4C8V) XP® Rabbit mAb	Cell Signaling Technology	Cat# 30325; RRID:AB_2798990
Human/Mouse TREM2 Allophycocyanin Mab	R&D systems	Cat# FAB17291A; RRID:AB_884527
Brilliant Violet 421™ anti-human CD206 Antibody	BioLegend	Cat# 141717; RRID:AB_2562232
PE/Cyanine7 anti-mouse CD11C Antibody	BioLegend	Cat# 117317; RRID:AB_493569
Brilliant Violet 711™ anti-mouse/human CD11B Antibody	BioLegend	Cat# 101242; RRID:AB_2563310
FITC anti-mouse CD45 Antibody	Thermo Fisher Scientific	Cat# 11-0451-82; RRID:AB_465050

**Chemicals, peptides, and recombinant proteins**		

GM6001	MedChemExpress	HY-15768
d-glucose	Sigma	G7021
TRIzol	Thermo Fisher Scientific	15596018
iQ SYBR Green Supermix	Bio-Rad	170-8884
Bovine serum albumin	Bioworld	22070008
Collagenase type I	Gibco	17100-017
anti-F4/80 MicroBeads	Miltenyi Biotec	130-110-443
PRO-PREP Protein Extraction Solution	iNtRON Biotechnology	17081
Protease inhibitor	Sigma	S8820
Phosphatase inhibitors	Roche	4906845001
Pierce™ BCA Protein Assay Kits	Thermo Fisher Scientific	23225
5× sample buffer	Elpis Biotech	EBA 1052
1640 Medium, HEPES	Gibco	22400089
2-Mercaptoethanol	Sigma	M3148
PMA	Sigma	79346
Dulbecco’s Modified Eagle Medium	Welgene	LM001-07
Fetal bovine serum	Gibco	16000044
Penicillin/streptomycin	Welgene	LS202-02
IBMX	Sigma	I5879
Dexamethasone	Cayman	11015
Insulin	Sigma	I9278
Brefeldin A	Biolegend	1499
Lipopolysaccharide	Sigma	L4391
Adenosine 5-triphosphate magnesium salt	Sigma	A9187
BODIPY 558/568 C12	Invitrogen	D3835
Vybrant™ DiO Cell-Labeling Solution	Invitrogen	V22886
10% formalin	Sigma	HT50128

**Critical commercial assays**		

soluble TREM2 (sTREM2) ELISA kit	Aviscera Bioscience	SK00218-30
High-Capacity cDNA Reverse Transcription Kit	Applied Biosystems	4368813

**Experimental models: Cell lines**		

RAW264.7	ATCC	Cat# TIB-71; RRID:CVCL_0493
3T3-L1	ATCC	ATCC Cat# CL-173; RRID:CVCL_0123
THP-1	ATCC	TIB-202

**Experimental models: Organisms/strains**		

C57/BL6J	The Jackson Laboratory	RRID:IMSR_JAX:000664

**Oligonucleotides**		

*Adam10-F*	Bionics	GTGCCAGTACAGGCTCTTTGC
*Adam10-R*	Bionics	CACAGTAGCCTCTGAAGTCATTACATG
*Adam17-F*	Bionics	AAGTGCAAGGCTGGGAAATG
*Adam17-R*	Bionics	CACACGGGCCAGAAAGGTT
*ADAM10-F*	Bionics	GAGGAGTGTACGTGTGCCAGTT
*ADAM10-R*	Bionics	GACCACTGAAGTGCCTACTCCA
*ADAM17-F*	Bionics	AACAGCGACTGCACGTTGAAGG
*ADAM17-R*	Bionics	CTGTGCAGTAGGACACGCCTTT
*Cd163-F*	Bionics	GGCTAGACGAAGTCATCTGCAC
*Cd163-R*	Bionics	CTTCGTTGGTCAGCCTCAGAGA
*Arg1-F*	Bionics	GGCTGGTGTGGTGGCAGAGG
*Arg1-R*	Bionics	CCTGGCGTGGCCAGAGATGC
*Il10-F*	Bionics	GGCAGAGAAGCATGGCCCAGAA
*Il10-R*	Bionics	TCACCTGCTCCACTGCCTTGC
*Ppia-F*	Bionics	GTGGTCTTTGGGAAGGTGAA
*Ppia-R*	Bionics	TTACAGGACATTGCGAGCAG
*PPIA-F*	Bionics	GGCAAATGCTGGACCCAACACA
*PPIA-R*	Bionics	TGCTGGTCTTGCCATTCCTGGA

**Deposited data**		

Raw bulk-RNA sequencing data	Gene Expression Omnibus	GSE86080, GSE182930, GSE280875
Mouse single-nucleus RNA-seq datasets	BioProject database	BioProject; accession number PRJNA942977
human single-cell/nucleus RNA-seq datasets	Gene Expression Omnibus	GSE176171

**Software and algorithms**		

LASX	LEICA	N/A
ImageJ	National Institute of Health	https://imagej.net/ij/
Prism v.9	GraphPad	https://www.graphpad.com
RStudio v.3.6.	Rstudio	https://satijalab.org/seurat/
Cytation 5	Agilient	N/A

### Experimental model and study participant details

#### Mice

Male C57BL/6J mice (6–16 weeks of age) were maintained under specific-pathogen-free conditions with a 12-hour light/dark cycle at 22 ± 1°C and *ad libitum* access to food and water. All experimental procedures were conducted in compliance with institutional and national ethical guidelines. Animal studies were approved by the Institutional Animal Care and Use Committee of Seoul National University (SNU-230130-5, SNU-250306-1) and were carried out in accordance with the guidelines for the humane care and use of laboratory animals provided by the Ministry of Food and Drug Safety.

#### Human sample

Human subcutaneous adipose tissue sample collection and analyses were performed in accordance with the Declaration of Helsinki and were approved by the Institutional Review Board of Korea University Guro Hospital (Institutional Review Board No. 2022GR0095). Informed consent was obtained from all participants. Clinical features of the patients are reported in Table S1. In particular, 15 males and 9 females of Asian ethnicity have been recruited (ages 9 – 79 years). No selection bias based on sex was intended.

#### Cell lines

RAW264.7 cells (ATCC, TIB-71) and 3T3-L1 cells (ATCC, CL-173) were authenticated by STR profiling. All cells incubated at 37°C with 5% CO2 and tested negative for mycoplasma contamination. Dulbecco’s Modified Eagle Medium (DMEM; Welgene, LM001-07) supplemented with 10% fetal bovine serum (FBS; Gibco, 16000044) and 1% penicillin/streptomycin (PS; Welgene, LS202-02) were used for growth medium. RAW264.7 cells (ATCC, TIB-71) were maintained in a growth medium until experiments were initiated. THP-1 cells (ATCC, TIB-202) were maintained in a RPMI medium (Gibco, 22400089) containing with 10% FBS and 0.05mM β-mercaptoethanol (Sigma, M6250). To differentiate THP-1 cells, 50nM of horbol 12-myristate 13-acetate (PMA; Sigma, P1585) was treated for 3 days. 3T3-L1 cells (ATCC, CL-173) were cultured in growth medium until confluency. Then the cells were then cultured in differentiation medium (growth medium containing IBMX (Sigma, 0.5 mM, I5879), dexamethasone (Cayman, 1 μM, 11015), insulin (Sigma, 10 μg/mL, I9278)) for 3 days, followed by exposure to maintenance medium (growth medium with insulin (10 μg/mL)) for an additional 3 days.

#### Primary cell culture

BMDMs were produced from femurs and tibias of 8-week-old of male mice under sterile conditions. Marrow cavities were flushed with DMEM using a syringe and 23G needle, and the resulting cell suspension was collected on ice. Cells were pelleted (500 × g, 5 min, 4 °C), resuspended in 1 mL of red blood cell lysis buffer, and centrifuged (500 × g, 5 min, 4 °C). After removal of the supernatant, cells were washed by BMDM medium consisting of growth medium supplemented with recombinant M-CSF (1 ng/mL; PeproTech, 315-02) and were filtered with filter through a 100 μm cell strainer. BMDMs were cultured in BMDM medium at 37 °C in 5% CO_2_. On day 3, cultures received a complete medium change with fresh BMDM medium, and differentiation was continued for an additional 3 days before phagocytosis assay or western blot analysis.

### Method details

#### Animal studies

For diet-induced obesity experiments, mice were fed a 60% high-fat diet (HFD; Research Diets, D12492) for up to 10 weeks, while control mice received standard rodent chow (NCD; Purina, 38057). GM6001 (7.5 mg/kg, MedChemExpress, HY-15768) was administered intraperitoneally at two-day intervals starting from week 4 of HFD feeding. Glucose tolerance test was performed after a 12-hour fast, followed by intraperitoneal injection of d-glucose (2 g/kg, Sigma, G7021). Blood glucose levels were assessed using tail vein blood with a GlucoDoctor Top meter (Allmedicus, AGM-4100), as previously described.^39^ Serum levels of soluble TREM2 (sTREM2) were quantified using a commercially available ELISA kit (Aviscera Bioscience, SK00218-30).

#### RNA extraction and quantitative PCR analysis

Total RNA was extracted from mouse and human adipose tissues using TRIzol reagent (Thermo Fisher Scientific, 15596018). Complementary DNA (cDNA) was synthesized with the High-Capacity cDNA Reverse Transcription Kit (Applied Biosystems, 4368814). Quantitative PCR was performed using iQ SYBR Green Supermix (Bio-Rad, 170-8884) on a Bio-Rad CFX Connect Real-Time PCR system. *Peptidylprolyl isomerase A* (*Ppia*) was used as a housekeeping gene and primers used for qPCR are listed in STAR Methods. 2-ΔCt method was used to calculate the relative expression levels of each gene.

#### Isolation of mature adipocytes and stromal vascular fraction (SVF)

Adipose tissues were dissected, minced, and digested in KRBB buffer containing 3% bovine serum albumin (BSA, Bioworld, 22070008) and 2 mg/mL collagenase type I (Gibco, 17100-017) at 37°C. The digests were filtered through a 100 μm cell strainer (Corning, 431752) and centrifuged at 300 × g for 5 min. Floating mature adipocytes were collected, and the remaining cell pellets were subjected to red blood cell lysis and washed twice. The SVF cells were isolated for magnetic-activated cell sorting (MACS) or cultured directly. For MACS, SVF cells were incubated with anti-F4/80 MicroBeads (Miltenyi Biotec, 130-110-443) at 4°C for 15 min and separated using magnetic columns. F4/80+ cells were collected and used for RNA extraction. For flow cytometry analysis, SVF cells were stained with fluorescence-labeled appropriate primary antibodies at room temperature, as previously described.^40^ Human/Mouse TREM2 Allophycocyanin Mab (FAB17291A, 1:50) were purchased from R&D systems. Brilliant Violet 421™ anti-human CD206 (141717, 1:100), PE/Cyanine7 anti-mouse CD11C (117317, 1:100), and Brilliant Violet 711™ anti-mouse/human CD11B (101242, 1:100) was purchased from Biolegend. FITC anti-mouse CD45 (11-0451-82, 1:100) was purchased from Invitrogen.

#### Western blot analysis

Western blot analysis was performed as previously described.^41^ Tissues (BAT, IWAT, GWAT) were homogenized in PRO-PREP Protein Extraction Solution (iNtRON Biotechnology, 17081) containing protease (Sigma, S8820) and phosphatase inhibitors (Roche, 4906845001) using a bead homogenizer. Protein concentration was determined by BCA assay (Thermo Fisher Scientific, 23225). Equal amounts (10 μg) of protein were denatured in 5 × sample buffer (Elpis Biotech, EBA 1052), resolved by SDS-PAGE, and transferred to PVDF membranes (Bio-Rad, 1620177). Membranes were blocked with 5% BSA in TBST and incubated with primary antibodies overnight at 4°C, followed by HRP-conjugated secondary antibodies for 1 h at room temperature. Antibodies used are listed in STAR Methods.

#### Phagocytosis assay

To produce apoptotic adipocytes, adipocytes were treated with Brefeldin A (1:1000, Biolegend, 1499) for 24 h. For pyroptotic adipocyte induction, adipocytes were treated with 100 ng/mL LPS (Sigma, L4391) for 48 h followed by 2 mM ATP (Sigma, A9187) for an additional 24 h. After treatment, dying adipocytes were trypsinized and transferred onto macrophages that had been pre-seeded one day prior at a 5:1 macrophage-to-adipocyte ratio. Macrophages were seeded in 6-well plates at a density of 5 × 10^5^ cells per well and cultured for 24 h in growth medium (DMEM supplemented with 10% fetal bovine serum (FBS) and 1% penicillin/streptomycin) before co-culture. Following 4 or 24 h of co-culture, the plates were gently rinsed with PBS to remove residual/remaining dying adipocytes and then subjected to western blot analysis. For phagocytosis analysis, co-culture experiments were performed as described above, with adipocytes stained using BODIPY 558/568 C12 (Invitrogen, D3835) and co-cultured with macrophages labeled with Vybrant™ DiO cell-labeling solution (Invitrogen, V22886) to enable visualization. To perform phagocytosis assay, macrophages were plated in 96-well plates (5 × 10^4^ cells per well) one day prior and maintained in growth medium. Dying adipocytes were harvested by trypsinization, added at a 1:5 adipocyte-to-macrophage ratio, and live-cell imaging was performed for 24 h. Live-cell imaging was conducted using the Operetta CLS system, Cytation 5, and analyzed with Harmony software and ImageJ software. For siRNA knockdown, BMDMs were transfected with 20 nM TREM2 siRNA (Bioneer, custom designed) and a negative control (Bioneer, SN-1003) for 48 h after differentiation.

#### Histology

IWAT and GWAT were fixed in 10% formalin (Sigma, HT50128), paraffin-embedded, and sectioned at 5 μm thickness. Sections were stained with hematoxylin (BBC Biochemical, MA010081) and eosin (BBC Biochemical, 3610) and visualized using Nikon Elements software (NIS BR 5.10.00). For immunostaining, paraffin sections were deparaffinized, rehydrated, and subjected to antigen retrieval using citrate buffer (pH 6.0). After blocking with 3% BSA in PBS, tissues were incubated with TREM2 and F4/80 antibody (Cell Signaling Technology, 30325, 1:200) and counterstained with DAPI. Six representative regions per sample were analyzed using a Leica TCS SP8 confocal microscope and LASX software.

### Quantification and statistical analysis

ImageJ software (NIH, version 1.52a) was used for densitometric quantification of immunoblots and histological images. Statistical analysis for snRNA-seq was performed using built-in function in Seurat with results presented in Figures 2 and 3. All other statistical analyses were performed using GraphPad Prism 9. The exact n values, statistical tests, and significance levels are provided in the corresponding figure legends. Quantitative data are presented as mean values ± SEM. For all experiments, n represents the number of biologically independent samples (mice, cells, or human patients): n = 3 – 6 mice or cells per group for western blot analyses; n = 3 mice per group for quantification of sTREM2 and serum triglyceride levels; n = 4 mice per group for flow cytometry and immunohistochemistry analyses; n = 6 mice per group for glucose tolerance test, body weight monitoring, and tissue weight measurement; and n = 12 human patients per group for correlation analysis of gene expression levels in subcutaneous adipose tissue. p-values were determined by the unpaired two-sided Student’s t-test.

## Supplemental information

Document S1. Figures S1–S5 and Table S1

Data S1. Original raw western blot files

## References

[1] Arner E., Westermark P.O., Spalding K.L., Britton T., Rydén M., Frisén J., Bernard S., Arner P. (2010). Adipocyte turnover: relevance to human adipose tissue morphology. Diabetes.

[2] Wentworth J.M., Naselli G., Brown W.A., Doyle L., Phipson B., Smyth G.K., Wabitsch M., O'Brien P.E., Harrison L.C. (2010). Pro-inflammatory CD11c+CD206+ adipose tissue macrophages are associated with insulin resistance in human obesity. Diabetes.

[3] Lumeng C.N., Bodzin J.L., Saltiel A.R. (2007). Obesity induces a phenotypic switch in adipose tissue macrophage polarization. J. Clin. Investig..

[4] Winn N.C., Wolf E.M., Garcia J.N., Hasty A.H. (2022). Exon 2-mediated deletion of Trem2 does not worsen metabolic function in diet-induced obese mice. J. Physiol..

[5] Jaitin D.A., Adlung L., Thaiss C.A., Weiner A., Li B., Descamps H., Lundgren P., Bleriot C., Liu Z., Deczkowska A. (2019). Lipid-Associated Macrophages Control Metabolic Homeostasis in a Trem2-Dependent Manner. Cell.

[6] Choi C., Jeong Y.L., Park K.M., Kim M., Kim S., Jo H., Lee S., Kim H., Choi G., Choi Y.H. (2024). TM4SF19-mediated control of lysosomal activity in macrophages contributes to obesity-induced inflammation and metabolic dysfunction. Nat. Commun..

[7] Wang X., He Q., Zhou C., Xu Y., Liu D., Fujiwara N., Kubota N., Click A., Henderson P., Vancil J. (2023). Prolonged hypernutrition impairs TREM2-dependent efferocytosis to license chronic liver inflammation and NASH development. Immunity.

[8] Hou J., Zhang J., Cui P., Zhou Y., Liu C., Wu X., Ji Y., Wang S., Cheng B., Ye H. (2021). TREM2 sustains macrophage-hepatocyte metabolic coordination in nonalcoholic fatty liver disease and sepsis. J. Clin. Investig..

[9] Patterson M.T., Firulyova M.M., Xu Y., Hillman H., Bishop C., Zhu A., Hickok G.H., Schrank P.R., Ronayne C.E., Caillot Z. (2023). Trem2 promotes foamy macrophage lipid uptake and survival in atherosclerosis. Nat. Cardiovasc. Res..

[10] Herrero L., Shapiro H., Nayer A., Lee J., Shoelson S.E. (2010). Inflammation and adipose tissue macrophages in lipodystrophic mice. Proc. Natl. Acad. Sci. USA.

[11] Lindhorst A., Raulien N., Wieghofer P., Eilers J., Rossi F.M.V., Bechmann I., Gericke M. (2021). Adipocyte death triggers a pro-inflammatory response and induces metabolic activation of resident macrophages. Cell Death Dis..

[12] Cinti S., Mitchell G., Barbatelli G., Murano I., Ceresi E., Faloia E., Wang S., Fortier M., Greenberg A.S., Obin M.S. (2005). Adipocyte death defines macrophage localization and function in adipose tissue of obese mice and humans. J. Lipid Res..

[13] Zhou Y., Song W.M., Andhey P.S., Swain A., Levy T., Miller K.R., Poliani P.L., Cominelli M., Grover S., Gilfillan S. (2020). Author Correction: Human and mouse single-nucleus transcriptomics reveal TREM2-dependent and TREM2-independent cellular responses in Alzheimer's disease. Nat. Med..

[14] Schlepckow K., Kleinberger G., Fukumori A., Feederle R., Lichtenthaler S.F., Steiner H., Haass C. (2017). An Alzheimer-associated TREM2 variant occurs at the ADAM cleavage site and affects shedding and phagocytic function. EMBO Mol. Med..

[15] Rayaprolu S., Mullen B., Baker M., Lynch T., Finger E., Seeley W.W., Hatanpaa K.J., Lomen-Hoerth C., Kertesz A., Bigio E.H. (2013). TREM2 in neurodegeneration: evidence for association of the p.R47H variant with frontotemporal dementia and Parkinson's disease. Mol. Neurodegener..

[16] Kleinberger G., Brendel M., Mracsko E., Wefers B., Groeneweg L., Xiang X., Focke C., Deußing M., Suárez-Calvet M., Mazaheri F. (2017). The FTD-like syndrome causing TREM2 T66M mutation impairs microglia function, brain perfusion, and glucose metabolism. EMBO J..

[17] Xiang X., Werner G., Bohrmann B., Liesz A., Mazaheri F., Capell A., Feederle R., Knuesel I., Kleinberger G., Haass C. (2016). TREM2 deficiency reduces the efficacy of immunotherapeutic amyloid clearance. EMBO Mol. Med..

[18] Yuan P., Condello C., Keene C.D., Wang Y., Bird T.D., Paul S.M., Luo W., Colonna M., Baddeley D., Grutzendler J. (2016). TREM2 Haplodeficiency in Mice and Humans Impairs the Microglia Barrier Function Leading to Decreased Amyloid Compaction and Severe Axonal Dystrophy. Neuron.

[19] Heslegrave A., Heywood W., Paterson R., Magdalinou N., Svensson J., Johansson P., Öhrfelt A., Blennow K., Hardy J., Schott J. (2016). Increased cerebrospinal fluid soluble TREM2 concentration in Alzheimer's disease. Mol. Neurodegener..

[20] Song W.M., Joshita S., Zhou Y., Ulland T.K., Gilfillan S., Colonna M. (2018). Humanized TREM2 mice reveal microglia-intrinsic and -extrinsic effects of R47H polymorphism. J. Exp. Med..

[21] Pugazhenthi S., Qin L., Reddy P.H. (2017). Common neurodegenerative pathways in obesity, diabetes, and Alzheimer's disease. Biochim. Biophys. Acta. Mol. Basis Dis..

[22] Dhandapani R., Neri M., Bernhard M., Brzak I., Schweizer T., Rudin S., Joller S., Berth R., Kernen J., Neuhaus A. (2022). Sustained Trem2 stabilization accelerates microglia heterogeneity and Abeta pathology in a mouse model of Alzheimer's disease. Cell Rep..

[23] Kothari V., Savard C., Tang J., Lee S.P., Subramanian S., Wang S., den Hartigh L.J., Bornfeldt K.E., Ioannou G.N. (2023). sTREM2 is a plasma biomarker for human NASH and promotes hepatocyte lipid accumulation. Hepatol. Commun..

[24] Hendrikx T., Porsch F., Kiss M.G., Rajcic D., Papac-Miličević N., Hoebinger C., Goederle L., Hladik A., Shaw L.E., Horstmann H. (2022). Soluble TREM2 levels reflect the recruitment and expansion of TREM2(+) macrophages that localize to fibrotic areas and limit NASH. J. Hepatol..

[25] Shirotani K., Hatta D., Wakita N., Watanabe K., Iwata N. (2022). The role of TREM2 N-glycans in trafficking to the cell surface and signal transduction of TREM2. J. Biochem..

[26] Emont M.P., Jacobs C., Essene A.L., Pant D., Tenen D., Colleluori G., Di Vincenzo A., Jørgensen A.M., Dashti H., Stefek A. (2023). Author Correction: A single-cell atlas of human and mouse white adipose tissue. Nature.

[27] Sui C., Zhou D. (2023). ADAM metallopeptidase domain 10 knockdown enables podocytes to resist high glucose stimulation by inhibiting pyroptosis via MAPK pathway. Exp. Ther. Med..

[28] Tsuchiya K., Hosojima S., Hara H., Kushiyama H., Mahib M.R., Kinoshita T., Suda T. (2021). Gasdermin D mediates the maturation and release of IL-1alpha downstream of inflammasomes. Cell Rep..

[29] Motani K., Kosako H. (2018). Activation of stimulator of interferon genes (STING) induces ADAM17-mediated shedding of the immune semaphorin SEMA4D. J. Biol. Chem..

[30] Decout A., Katz J.D., Venkatraman S., Ablasser A. (2021). The cGAS-STING pathway as a therapeutic target in inflammatory diseases. Nat. Rev. Immunol..

[31] Wang S., Sudan R., Peng V., Zhou Y., Du S., Yuede C.M., Lei T., Hou J., Cai Z., Cella M. (2022). TREM2 drives microglia response to amyloid-beta via SYK-dependent and -independent pathways. Cell.

[32] Menikdiwela K.R., Ramalingam L., Abbas M.M., Bensmail H., Scoggin S., Kalupahana N.S., Palat A., Gunaratne P., Moustaid-Moussa N. (2020). Role of microRNA 690 in Mediating Angiotensin II Effects on Inflammation and Endoplasmic Reticulum Stress. Cells.

[33] Zatterale F., Longo M., Naderi J., Raciti G.A., Desiderio A., Miele C., Beguinot F. (2019). Chronic Adipose Tissue Inflammation Linking Obesity to Insulin Resistance and Type 2 Diabetes. Front. Physiol..

[34] Park M., Yi J.W., Kim E.M., Yoon I.J., Lee E.H., Lee H.Y., Ji K.Y., Lee K.H., Jang J.H., Oh S.S. (2015). Triggering receptor expressed on myeloid cells 2 (TREM2) promotes adipogenesis and diet-induced obesity. Diabetes.

[35] Zhong L., Chen X.F., Wang T., Wang Z., Liao C., Wang Z., Huang R., Wang D., Li X., Wu L. (2017). Soluble TREM2 induces inflammatory responses and enhances microglial survival. J. Exp. Med..

[36] Ghiarone T., Castorena-Gonzalez J.A., Foote C.A., Ramirez-Perez F.I., Ferreira-Santos L., Cabral-Amador F.J., de la Torre R., Ganga R.R., Wheeler A.A., Manrique-Acevedo C. (2022). ADAM17 cleaves the insulin receptor ectodomain on endothelial cells and causes vascular insulin resistance. Am. J. Physiol. Heart Circ. Physiol..

[37] Menghini R., Fiorentino L., Casagrande V., Lauro R., Federici M. (2013). The role of ADAM17 in metabolic inflammation. Atherosclerosis.

[38] Saad M.I., Jenkins B.J. (2024). The protease ADAM17 at the crossroads of disease: revisiting its significance in inflammation, cancer, and beyond. FEBS J..

[39] Kim S., Choi C., Son Y., Lee J., Joo S., Lee Y.H. (2025). BNIP3-mediated mitophagy in macrophages regulates obesity-induced adipose tissue metaflammation. Autophagy.

[40] Lee Y.H., Petkova A.P., Granneman J.G. (2013). Identification of an adipogenic niche for adipose tissue remodeling and restoration. Cell Metab..

[41] Cho Y.K., Son Y., Kim S.N., Song H.D., Kim M., Park J.H., Jung Y.S., Ahn S.Y., Saha A., Granneman J.G., Lee Y.H. (2019). MicroRNA-10a-5p regulates macrophage polarization and promotes therapeutic adipose tissue remodeling. Mol. Metab..

