# Supplementary material for: Obesity-induced pyroptotic adipocyte death leads to TREM2-dependent macrophage dysfunction and adipose tissue inflammation
Source: iScience. 2025 Dec 6;29(1):114358. doi: 10.1016/j.isci.2025.114358 (PMC12775877; doi:10.1016/j.isci.2025.114358)
Supplement: Document S1. Figures S1–S5 and Table S1 [file mmc1.pdf]

iScience, Volume *29*

Supplemental information

Obesity-induced pyroptotic adipocyte death leads to TREM2-dependent macrophage dysfunction and adipose tissue inflammation

Cheoljun Choi, Junhyuck Lee, Gyeongran Park, Sik Namgoong, and Yun-Hee Lee

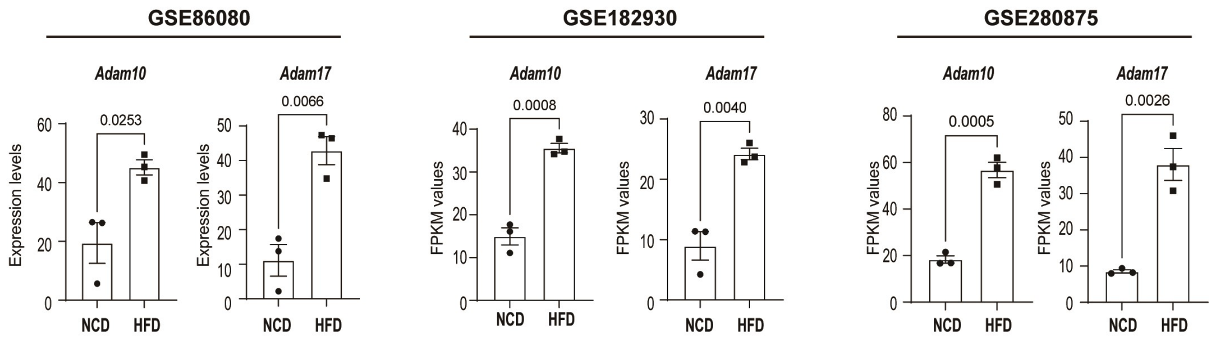

Figure S1. High-fat diet feeding increases *Adam10* and *Adam17* expression levels in GWAT, related to Figure 2.

Transcriptomic analysis of *Adam10* and *Adam17* expression levels in gonadal white adipose tissue (GWAT), determined by publicly available transcriptomic analyses (Gene Expression Omnibus (GEO) repository, accession numbers GSE86080, GSE182930, GSE280875). (n = 3 mice per group)

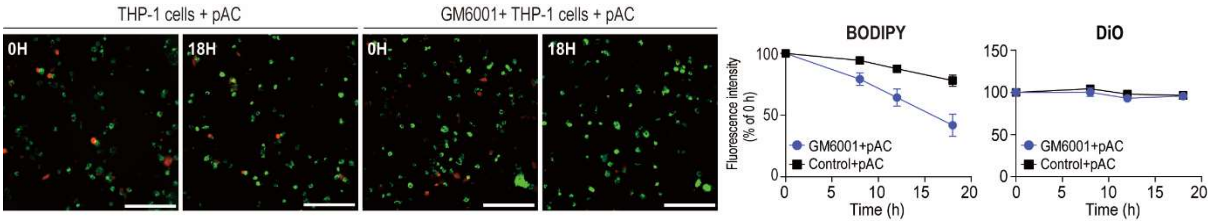

Figure S2. GM6001 increases the phagocytic activity of pyroptotic adipocytes in THP-1 cells, related to Figure 5.

Phagocytosis analysis of THP-1 cells co-cultured with pyroptotic adipocytes (pAC) for 18 h in the presence of GM6001. Adipocytes were tagged with C12-BODIPY (red), and THP-1 cells were stained with DiO (Green). Representative images from three independent experiments are shown, with quantification provided in the right panel. Scale bar = 200 μm.

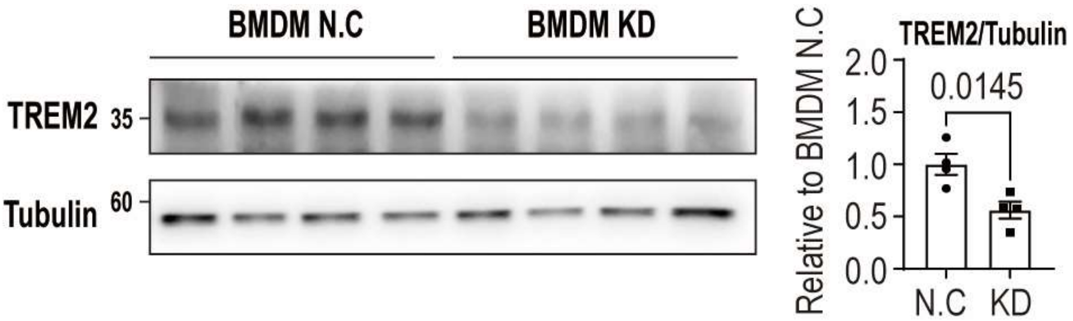

Figure S3. Confirmation of TREM2 knockdown (KD) in mouse bone marrow-derived macrophages, related to Figure 6.

Immunoblot analysis of TREM2 protein expression levels in mouse BMDMs after transfecting with TREM2 siRNA and negative control (n.c.) (20 nM). (n = 4 cells per group)

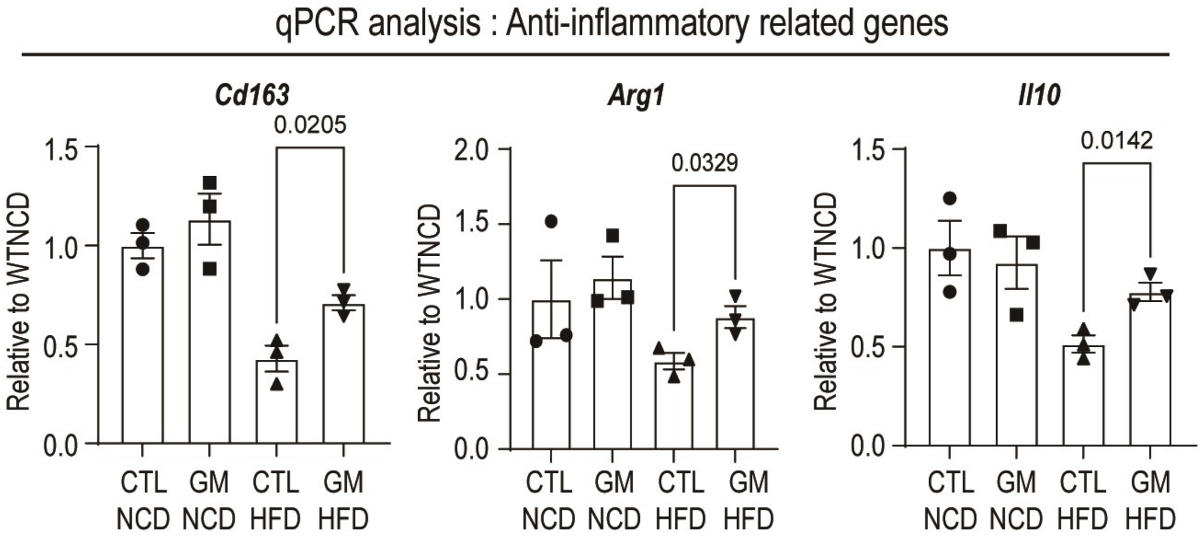

Figure S4. GM6001 treatment restores anti-inflammatory genes in GWAT of HFD-fed mice, related to Figure 7.

qPCR analysis of anti-inflammatory related genes levels (*Cd163, Arg1, Il10*) in GWAT of GM6001-treated mice after 10 weeks of HFD feeding (n = 3 mice per group).

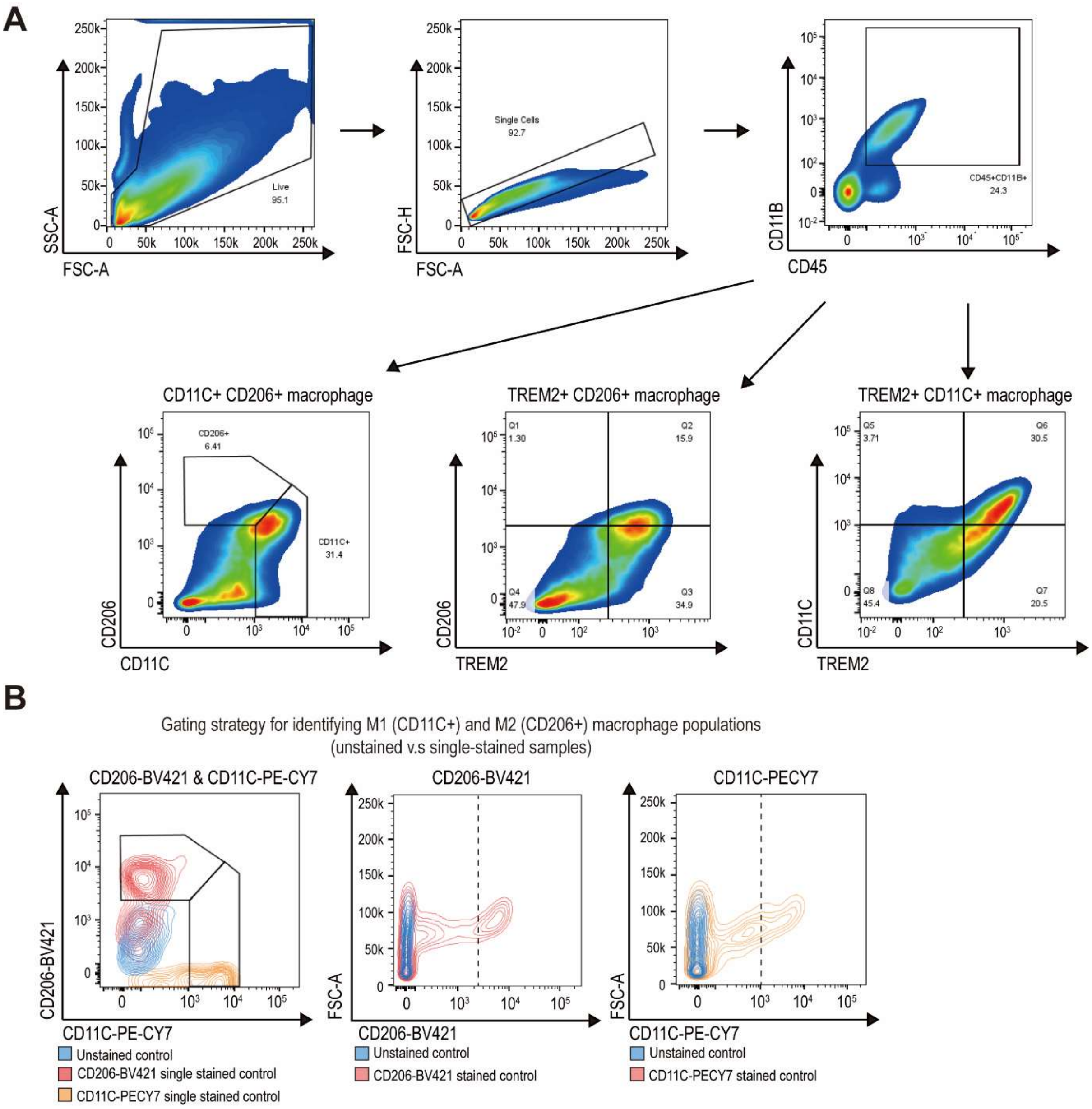

Figure S5. Flow cytometry gating strategy, related to Figure 7.

A. Representative flow profiles of the gating strategy of stromal vascular fraction (SVF) cells in GWAT of GM6001-treated mice after HFD feeding for 10 weeks.

B. Overlay contour plots of unstained and single-stained controls for CD11C and CD206, used to define gating boundaries in flow cytometry.

Table S1. Population characteristics of human subcutaneous adipose tissue samples, related to STAR★Methods.

**Variable**	
Age, *y*	9 ~ 79
Male, *n* (%)	62.5
Female, *n* (%)	37.5
BMI, kg/m^2	19.11 ~ 32.38
Height, *m*	138.6 ~ 178
Weight, *kg*	40.8 ~ 91.2
